# Effectiveness of a novel chelating agent in removing calcium hydroxide using conventional and passive ultrasonic irrigation techniques

**DOI:** 10.4317/jced.60782

**Published:** 2023-10-01

**Authors:** Mehmet-Omer Gorduysus, Melahat Gorduysus, Lovely-Muthiah Annamma

**Affiliations:** 1 DDS,PhD, Professor, Department of Preventive and Restorative Dentistry, College of Dental Medicine, University of Sharjah, United Arab Emirates; 2 DDS, PhD, Professor in Endodontics, Private Practice, Apollon Dental Center, Antalya, Türkiye; 3 BDS, PhD, Assistant Professor, Department of Clinical Sciences, College of Dentistry, Ajman University, United Arab Emirates; 4 Assistant Professor, Center of Medical and Bio-Allied Health Sciences Research, Ajman University, Ajman, United Arab Emirates

## Abstract

**Background:**

The present *in vitro* study aimed to compare the efficacy of a 25% copolymer of acrylic acid and maleic acid [poly(AA-co-MA)] and 17% of ethylenediaminetetraacetic acid (EDTA) in removing calcium hydroxide (CH) from root canals using the master apical file (MAF) and passive ultrasonic irrigation (PUI).

**Material and Methods:**

Fifty-eight teeth were dressed with CH. After 10 days, CH was removed using MAF+EDTA, MAF+poly(AA-co-MA), PUI+EDTA, and PUI+poly (AA-co-MA) (n=12). Ten teeth were used as controls. Residual CH was evaluated using a four-grade scoring system. Data analysis was performed using the Mann-Whitney U, Friedman, and Dunn-Bonferroni tests.

**Results:**

In the MAF and PUI groups, there was no significant difference in the CH scores between EDTA and Poly(AA-co-MA) (*p*<0.083). Although EDTA and poly(AA-co-MA) had lower CH scores when used with PUI, no significant difference was found between the two agents (*p*<0.083).

**Conclusions:**

Poly(AA-co-MA) did not remove significantly more CH than EDTA when used with MAF or PUI.

** Key words:**Calcium hydroxide, Ethylenediaminetetraacetic acid, Acrylic acid, Maleic acid.

## Introduction

The main goal of endodontic therapy is to eliminate microorganisms from infected root canal systems and prevent re-infection (1). Biological and antimicrobial properties make calcium hydroxide (CH) the most commonly used intracanal dressing material in endodontics (2).

CH is highly effective against the majority of strains identified in root canal infections (2). However, it needs to be effectively removed from the root canal prior to root canal obturation to prevent any possible negative effect on treatment. Residual CH compromises the quality of the seal by prohibiting sealer penetration into the dentin tubules (3), and leakage may occur over time due to the dimensional instability and solubility of this compound (3). It also creates weak bond strengths between dentin and filling materials (4), and it reacts with the sealer, reducing its flow and working time (5). Moreover, residual CH impairs the accuracy of electronic apex locators (6).

The most frequently described method for the removal of CH is mechanical instrumentation of the root canal with the master apical file (MAF) in combination with copious irrigation with sodium hypochlorite (NaOCl) and ethylenediaminetetraacetic acid (EDTA) (7,8). Conventional needle irrigation with a syringe is still widely used for irrigation delivery (7). However, complete removal of all of the CH has been found to be impossible using MAF and needle irrigation (9,10). Irregularities or complexity of the root canal system may be unreachable for conventional irrigation techniques, and CH may persist in these areas (9).

To date, studies have been conducted on the removal of CH utilizing different products and devices, such as sonic activation, passive ultrasonic irrigation (PUI), laser-activated irrigation, canal brush systems, nickel-titanium rotary instruments, apical negative pressure irrigation, and the RinsEndo system (11-16). Regardless of the method used, the general consensus is the creation of mechanical agitation (17-19). Ultrasonic agitation of irrigants is widely accepted by endodontists (18-20). PUI uses an ultrasonically activated file inside the root canal with a continuous irrigant. Previous studies have shown that PUI is more effective in removing CH from the root canal walls than delivery of the irrigant by positive pressure (13) or photon-induced photoacoustic streaming (16).

Apart from irrigation methods, several irrigation solutions, including EDTA and its derivatives, phosphoric acid, maleic acid, and citric acid, have been investigated to improve CH removal from root canals (8,17,21). These solutions have the capacity to chelate CH residues and make them easier to eliminate by irrigation (18). However, none of the described techniques and irrigation protocols seem to be able to completely remove CH from the root canal, which remains a challenge (18). The complexity of the root canal system, type of CH, irrigation times, irrigation solutions, and their concentration are crucial factors that can influence the efficacy of CH removal techniques. In addition, according to several reports, using EDTA and NaOCl in a sequential order can cause dentinal erosion on the root canal wall (22,23).

Recently, it has been shown that poly(AA-co-MA), obtained through the radical copolymerization of maleic anhydride (MA) with acrylic comonomers, is effective in removing the smear layer and debris without causing erosion on the dentin wall (24). This copolymer is regarded as having good chelating properties and is also biocompatible (25). However, poly(AA-co-MA) is a novel chelator, and its CH removal capacity remains unknown.

The hypotosis is that the poly (AA-co-MA) might be more effective for removing CH from the canal system than EDTA. Therefore, the current study aimed to evaluate the efficacy of different irrigation techniques and this novel chelator in removing CH from three different parts of the root canal system.

## Material and Methods

-Selection and preparation of teeth:

Fifty-eight extracted human maxillary incisor teeth with a 0-10° curvature were used. Teeth with more than a single canal and apical foramen, immature root apices, cracks and/or fractures on the root surface, and internal or external resorptions were excluded from the study.

Each tooth was decoronated at 16 mm from the apex to standardize the length of the roots. Patency was confirmed with a size 10 K-file (Dentsply Maillefer, Ballaigues, Switzerland), and the working length (WL) was determined to be 1 mm short of the apical foramen. All canals were instrumented using ProTaper (Dentsply-Maillefer Ballaigues, Switzerland) rotary instruments in a crown-down manner, according to the manufacturer’s recommendations. Each canal was prepared up to an F4 (#40) finishing file, and among each instrumentation, a size 10 K-file was used to confirm apical patency. The root canals were irrigated with 2 mL of 2.5% NaOCl (Sultan, WA, USA) after the use of each instrument.

Final irrigation was made with 5 mL of 2.5% NaOCl for 1 minute, followed by 5 mL of 17% EDTA (Vista Dental Products, Racine, WI, USA) for 1 minute. Subsequently, the root canals were rinsed with 10 mL of distilled water to remove any residual solution remaining in the canal, and then dried with paper points. Five specimens were used as a negative control group.

Chemically pure (95%) CH powder (Merck KGaA, Darmstadt, Germany) was mixed with distilled water at a powder to liquid ratio of 1:1, and the root canals in each group, except the negative control group, were filled with CH paste using a #30 Lentulo spiral (Mani Inc., Tochigi, Japan) with a slow-speed handpiece until the material extruded through the apex. Mesio-distal and bucco-lingual radiographs were taken to confirm complete root canal filling with CH.

The access cavities were temporarily sealed with a cotton pellet and a temporary filling material (Cavit Espe, Seefeld, Germany), and then the specimens were stored at 100% humidity at 37 °C for 10 days. Five specimens were served as a positive control group, in which CH removal was not performed.

All specimens were prepared by the same operator under standardized conditions.

-Removal of CH

After the root canals were prepared and obturated with CH, the remaining specimens were randomly divided into four experimental groups (n = 12) according to the CH removal techniques as follows:

MAF + EDTA: The root canals were filed manually with a size 40 Hedstrom file in a circumferential filing action for 1 minute, and a final flush of 5 mL of 2.5% NaOCl, 5 mL of 17% EDTA for 1 minute, using manual needle irrigation. The Endo-Eze needle (Endo-Eze, Ultradent, South Jordan, UT, USA) was used with a syringe. The 27-G side-vented needle was placed into the canal 2 mm shorter than the WL without any binding. During the delivery of the irrigation solution, the needle was moved 1-2 mm up and down to produce agitation and prevent binding or wedging of the needle.

MAF + poly(AA-co-MA): The root canals were filed manually with a size 40 Hedstrom file in a circumferential filing action for 1 minute in the same manner. Final irrigation was performed as described above using EDTA and 5 mL of 25% poly(AA-co-MA) (Aldrich Chemical Co., Milwaukee, WI, USA) for 1 minute with the Endo-Eze needle.

PUI + EDTA: A 15-K file (Dentsply, Maillefer) was placed on the WL to loosen CH and create space for the irrigation tips. Then, final irrigation was performed with an ultrasonic device (Suprasson Pmax Satelec, Acteon, Marignac, France) using a size 15 file (Irrisafe K 15 Satalec, Marignac, France). The Irrisafe instrument was placed 1 mm shorter than the WL and activated. The root canals were irrigated copiously with the ultrasonic activation of a vibrating file at power setting 5 using 5 mL of 2.5% NaOCl, 5 mL of 17% EDTA, and 5 mL of distilled water for 1 minute.

PUI + poly(AA-co-MA): Final irrigation was performed with PUI as described above. Instead of EDTA, 5 mL of poly(AA-co-MA) was used for 1 minute.

After each procedure, the root canals were rinsed with 5 mL of distilled water to remove any residual solution remaining in the canal, and then dried with paper points.

-Preparation of Samples:

Residual CH evaluation.

Following final irrigation, grooves were prepared with a water-cooled diamond bur on the buccal and lingual surfaces of the specimens and were split into two halves along the long axis buccolingually using a surgical chisel. The appropriate half of each root with a visible semi-canal lumen and more CH remnants was selected. Evaluation samples were sectioned into apical, middle, and coronal thirds by marking grooves at the root margins and digitally photographed using a digital camera mounted on a stereomicroscope (Leica DFC 280, Leica Microsystems, Wetzlar). Evaluations were made for each of the coronal, middle, and apical thirds of the root canal surfaces for each specimen at ×16 magnification.

Residual CH was scored blindly by two endodontists after a calibration exercise. The Kappa test was performed for CH evaluations to verify inter-observer agreement. CH scoring was performed using the four-grade scale developed by Lambrianidis *et al*. (8) : score 1, no visible remnants; score 2, scattered remnants; score 3, distinct masses; and score 4, densely packed remnants.

Three randomly selected specimens in each group were prepared for scanning electron microscopy (SEM; JEOL JSM-6400, Tokyo, Japan) analysis to represent residual CH and surface micromorphology. The selected specimens were dehydrated by a series of graded ethanol solutions, coated with a gold layer, and then evaluated using SEM at ×1,000 and 1,500 magnifications.

-Statistical Analysis:

Cohen’s kappa coefficients (κ) were calculated to determine the degree of inter-observer agreement levels in terms of calcium scores. Data were analyzed using IBM SPSS Statistics ver. 25 (IBM Corporation, Armonk, NY, US) software. Descriptive statistics were displayed as numbers (n) and percentages (%) or median (25th-75th percentile) values, where applicable. The Mann-Whitney U test was applied for the comparison of the CH scores according to the irrigation techniques and chelating agents used. The statistical significance of differences in the CH scores among localizations within each irrigation technique and chelating agent pair was examined with the Friedman test. When the *p-value*s from the Friedman test were statistically significant, the Dunn-Bonferroni test was conducted to identify the localization that significantly differed from the others. A *p* value of less than 0.05 was considered statistically significant. For each possible multiple comparison, the Bonferroni correction was applied to control type I error.

## Results

There was substantially high agreement between the examiners in terms of the CH scores at coronal, middle, and apical third levels of the root canal (κ: 0.770, *p* < 0.001; κ: 0.775, *p* < 0.001; and κ: 0.746, *p* < 0.001, respectively).

**Figure 1:**
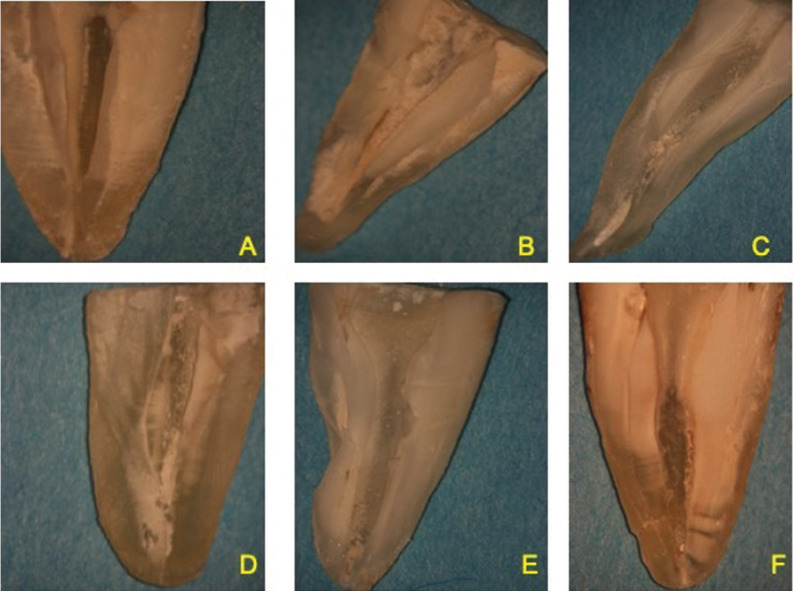
Representative stereomicroscopic images of all groups with the residual CH for the apical, middle, and coronal thirds of the root canal: A) negative control, B) positive control group, C) MAF + EDTA group, D) MAF + poly(AA-co-MA) group, E) PUI + EDTA group, and F) PUI + poly(AA-co-MA) group.

**Figure 2:**
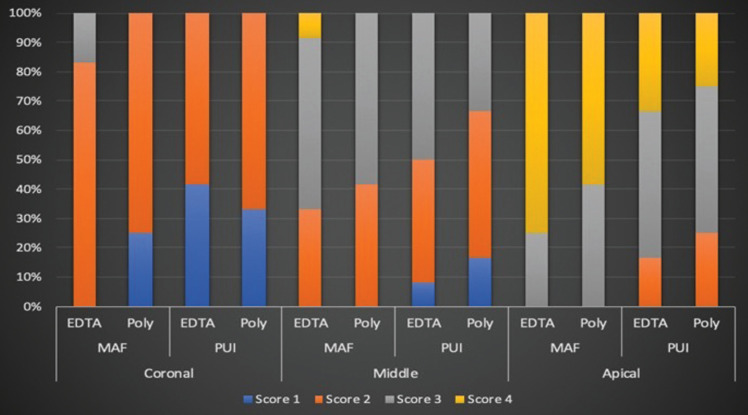
Distribution of the residual CH scores of the groups.

**Figure 3:**
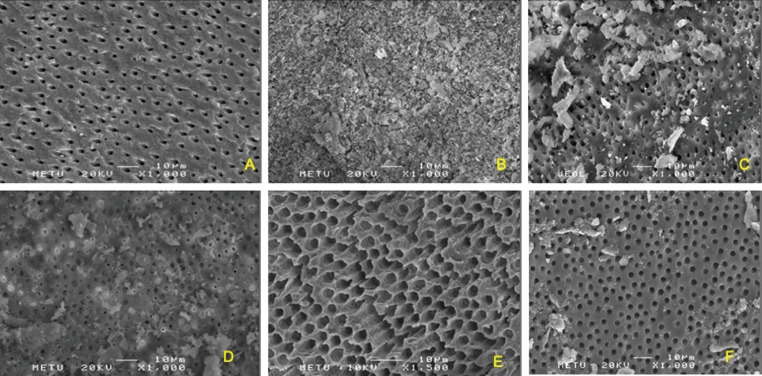
Representative SEM images of the middle third of the root canal in all groups: A) negative control group (intact dentin tubules), B) positive control group (fully covered with CH; not evaluated), C) MAF + EDTA (residual CH and intact dentin tubules), D) MAF + poly(AA-co-MA) group, (intact dentin, score: 2) E) PUI + EDTA group (severe erosion), and F) PUI + poly(AA-co-MA) group (intact dentin, score: 2).

Representative stereomicroscopic images of all groups with their scores are shown in Figure 1.The negative control group had no residual CH on the apical, middle, and coronal thirds of the root canal (scores: 1 for all) (Fig. 1A), while the positive control group showed that the canal walls were completely filled with CH (scores: 4 for all) (Fig. 1B). The CH scores of the experimental groups for the apical, middle, and coronal thirds were as follows: 4, 2, and 2, respectively, in the MAF+EDTA group (Fig. 1C); 4, 3,and 2, respectively, in the MAF + poly(AA-co-MA) group (Fig. 1D); 3, 2, and 1, respectively, in the PUI + EDTA group (Fig. 1E); and 2, 1, and 1, respectively, in the PUI + poly(AA-co-MA) group (Fig. 1F).

The statistical results of the CH scores between each third of the root canal (coronal, middle, and apical) within and between the groups are shown as median (25th-75th percentile) values in Table 1. There were no significant differences between EDTA and poly(AA-co-MA) in terms of the CH scores at the coronal, middle, and apical thirds of the canal in the evaluation of the MAF groups (*p* = 0.128, *p* = 0.590, and *p* = 0.514 respectively) or the PUI groups (*p* = 0.755, *p* = 0.443, and *p* = 0.630, respectively) (Table 1).

**Table 1:**
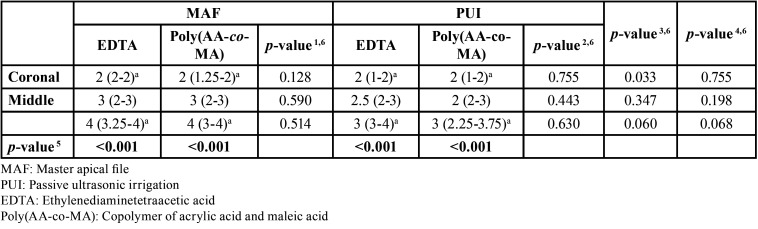
Comparisons of the residual CH scores between the experimental groups. Data were shown as median (25th-75th percentile) values. 1Comparisons between the two agents for each third of the root canal within the MAF groups, 2Comparisons between the two agents for each third of the root canal within the PUI groups, 3Comparisons between the two irrigation methods for each third of the root canal within the EDTA groups, 4Comparisons between the two irrigation methods for each third of the root canal within the poly(AA-co-MA) groups, 5Comparisons between the apical, middle, and coronal thirds of the root canal according to irrigation and agent combinations (the Friedman test was used, and a *p*-value less than 0.0125 according to the Bonferroni correction was considered statistically significant), 6Mann-Whitney U test was used, and according to the Bonferroni correction, a p-value less than 0.0083 was considered statistically significant. aIndicates statistically significant differences between the coronal and apical thirds of the root canal (*p* <0.01).

For the samples in which EDTA was used, although the PUI group resulted in a lower CH score (better result) in the coronal, middle, and apical thirds of the root canal, the differences were not statistically significant according to the Bonferroni correction (*p* = 0.033, *p* = 0.347, and *p* = 0.060, respectively). The same result was observed in the poly(AA-co-MA)-used samples (*p* = 0.755, *p* = 0.198, and *p* = 0.068, respectively) (Table 1).

Residual CH with a high score was observed at the apical third in all experimental groups. The PUI groups had lower CH scores in the apical and middle thirds of the root canal, regardless of the chelating agent used. While score 2 was more frequent in all experimental groups, score 1 was more frequent in the PUI groups. Scores 3 and 4 were observed at a higher rate for the apical third of the root canal in the MAF groups, regardless of the irrigant (Fig. 2, Table 1).

Regardless of the removal technique, there were significant differences between the apical and coronal thirds of the root canal in terms of the CH scores (*p* < 0.001). However, no statistically significant difference was found between the remaining paired comparisons according to the Bonferroni correction (*p* > 0.0125) (Table 1).

Representative stereomicroscopic images of all groups with their scores are shown in Figure 1.

Representative SEM images of the middle third of the experimental and control groups are shown in Figure 3. The Figures were taken from the middle third of the root canal to allow for the evaluation of surface micromorphology. The erosive effect of EDTA was seen in the PUI groups (Fig. 3E). Intact dentin tubules were seen in the negative control (Fig. 3A) and PUI + poly(AA-co-MA) groups (Fig. 3F), while the positive control was fully covered with CH (not possible to evaluate erosion) (Fig. 3B), the MAF + EDTA group had a score of 2 with intact dentin tubules (Fig. 3C), and the MAF + poly(AA-co-MA) group also had a score of 2 (Fig. 3D).

## Discussion

The removal of CH medicament before obturation is crucial, and to date, a wide range of techniques and solutions have been used for this purpose. The aim of this study was to evaluate the efficacy of different irrigation techniques and a novel chelator for CH removal from the root canals.

In the current study, none of the groups evaluated were able to completely remove CH from the root canal. This is consistent with the results of previous studies showing residues on the root canal walls, regardless of the removal technique used (8,10,17). On the other hand, both poly(AA-co-MA) and EDTA had lower scores in the PUI group than in the MAF group for each third of the root canal.

We conducted evaluations according to each third of the root canal separately due to the likely unequal spread of residual CH at different root levels. According to the results of the study, the CH scores were higher in the middle third regardless of the method used, contrary to the literature (26,27). This may be because unlike previous studies (8,10,11,13), half of the root with higher amounts of CH residue was evaluated, with the other half not being included in the evaluation. Another reason may be related to the smaller number of samples. In terms of scoring in the coronal third of the root canal, there were no significant differences between the MAF and PUI groups combined with EDTA or poly(AA-co-MA).

Residual CH in the canal is calculated by several methods, such as scoring systems, SEM, stereomicroscopy, micro-computed tomography, and confocal laser scanning microscopy (8,16,26-28). In the current study, stereomicroscopic images were obtained, and a scoring system was used, as previously described in the literature (6,14). Stereomicroscopic images were scored by two independent evaluators who had substantially high agreement in terms of the CH scores at the coronal, middle, and apical third levels. Due to the color similarity between CH and various dentin parts, it is difficult to automatically select residually covered areas using the proper software. Therefore, contrary to previous studies (29), in the current study, the percentage ratio of the CH-coated surface area was not calculated. Scoring system was considered reliable.

EDTA has the capacity to chelate CH residues, which could prevent an interaction with the sealer, making it easier to remove CH from the root canal. However, it has been reported that EDTA may lead to dentinal erosion on the root canal wall, especially when used with NaOCl and/or agitation (21,22). Erosion may change the mechanical characteristics of dentin and make it more challenging for root filling materials to adapt to canal walls (30). It has been shown that citric acid and EDTA have erosive effects on the dentin surface when used with increasing agitation (16).

Poly(AA-co-MA) is a polymer with numerous applications in the biomedical area. There are also reports of the use of this agent in pharmaceutical drugs, drug conjugates, enzyme-conjugates, or gene delivery systems (31). Poly(AA-co-MA) has chelating properties similar to EDTA and can increase mucosal permeability in pharmaceutical forms (31). It has been shown that this copolymer has promising results as a novel and potent irrigant, producing entirely clean dentin surfaces without damaging the dentinal surface (24).

In the current study, the SEM evaluation was performed on the middle third of the root canal. In the positive control group, the apical third of the root canal was fully covered with CH, which did not allow for the evaluation of the micromorphology of the dentinal surface. However, the SEM images cannot be considered the primary data set of the current study. While normal dentin surfaces were observed in the negative control group, dentin erosion was observed only in the PUI-agitated EDTA group.

Before the invention of PUI, conventional irrigation with syringes was recommended as an effective method of irrigant administration. MAF with syringe irrigation is still a widely accepted method for the removal of CH medicament by both general practitioners and endodontists. This is the reason why both techniques (MAF and PUI) were used in the current study. In the MAF groups, the irrigant was dispensed into a canal with agitation in a circumferential filing manner by moving the needle up and down. In these groups, no significant differences were observed between EDTA and poly(AA-co-MA) in terms of the CH scores at the coronal, middle, or apical thirds of the root canal.

Previous studies have found that CH intracanal medicament can be effectively removed from root canals using PUI with chelators (9,16).This may be due the synergistic effect of chelators with acoustic streaming and cavitation. Lee *et al*. (9) compared PUI and syringe irrigation and achieved more dentin debris removal with the former through microstreaming and cavitation within the root canal. Later, Van der Suis *et al*. (18) compared PUI and syringe irrigation in terms of CH removal and reported that PUI irrigation resulted in more dentin debris removal via the same mechanisms. If cavitation cannot be created, air trapped in the apical region causes a vapor lock and block, which prevents fluid flow and exchange. This can also explain the higher CH scores obtained from the middle third of the root canal. Furthermore, the irrigants are only delivered up to 1 mm from the needle tip, making it impossible to flush the apical third of the root canal. Syringe irrigation also delivers an insufficient volume of irrigants to the apical area and lacks cavitation, which may result in low efficacy in CH removal. However, Balvedi *et al*. (32), who compared syringe injection and PUI for the removal of CH from root canals, found no significant difference between these two methods.

The volume, concentration, duration, and delivery method of chelators can affect CH removal. Moreover, the methodology used and the type and placement of CH (longitudinal artificial grooves or intact root canals) can also affect the success of CH removal.

## Conclusions

In this study, poly(AA-co-MA) did not remove significantly more CH from the root canal than EDTA when used with MAF or PUI. Nevertheless, PUI had lower CH scores in both the MAF and PUI groups. In order to generalize results to clinical scenarios, there is a need for further *in vivo* and *in vitro* investigations with larger samples.
